# Effects of 12 months aerobic exercise intervention on work ability, need for recovery, productivity and rating of exertion among cleaners: a worksite RCT

**DOI:** 10.1007/s00420-017-1274-3

**Published:** 2017-11-04

**Authors:** Mark Lidegaard, Karen Søgaard, Peter Krustrup, Andreas Holtermann, Mette Korshøj

**Affiliations:** 1 Centre for Ergonomics, Occupational Safety and Health, School of Health Sciences, College of Health, Massey University, Palmerston North, New Zealand; 2 Department of Sports Science and Clinical Biomechanics, University of Southern Denmark, Odense, Denmark; 3 Department of Clinical Research, University of Southern Denmark, Odense, Denmark; 4 Sport and Health Sciences, College of Life and Environmental Sciences, University of Exeter, Exeter, UK; 5 National Research Centre for the Working Environment, Copenhagen, Denmark

**Keywords:** Work demands, Occupational health, Blue-collar workers

## Abstract

**Purpose:**

This study assessed the effects of a worksite aerobic exercise intervention among cleaners on: work ability, need for recovery, productivity, and rating of exertion.

**Methods:**

In a monocentric randomised controlled trial in Denmark, 116, of 250 invited, cleaners were cluster-randomised (work location; sex; age; length of service) to aerobic exercise [*N* = 57, 44.9 years, 75.4% female, body mass index (BMI) 26.2], receiving 2 weekly aerobic exercise sessions during 12 months, or a reference group (*N* = 59, 45.7 years, 76.3% female, BMI 27.1), receiving health-promoting lectures. Self-reported data on outcomes and sociodemographic information were collected at baseline, and at 4 and 12 month follow-up. All outcomes were analysed in a linear repeated-measures 2 × 2 mixed-model by an intention-to-treat analysis approach.

**Results:**

Drop-out was 26 and 33% at 4 and 12 months, respectively. Aerobic exercise adherence was 51% during the first 4 months. At 4 month follow-up no effects were found. At 12 month follow-up, work ability significantly increased by 0.59 on a 0–10 scale (95% CI 0.05–1.13) and need for recovery significantly decreased by − 11.0 on a 0–100 scale (95% CI − 19.8 to − 2.2) in the aerobic exercise group compared to the reference group. Productivity and rating of exertion were unaltered. Analysis stratified on age showed significant effects only among the participants aged ≤ 45 years.

**Conclusions:**

After 12 months work ability improved and need for recovery decreased. A period of 4 months was insufficient to affect these outcomes emphasising that longer interventions may be needed to induce effects on work ability and need for recovery.

## Introduction

The risk of health problems and early retirement increases when the work demands are greater than the mental or physical capacity of the worker (Armstrong et al. 1993). Cleaners represent an occupational group with well-documented high physical work demands (Søgaard et al. 2006), such as long periods of standing and walking (Korshøj et al. 2013), substantial time with static workloads (Søgaard et al. 2006), as well as awkward postures like forward bending and work with arms above shoulder level (Krüger et al. 1997). Furthermore, cleaners have a low cardiorespiratory fitness (Korshøj et al. 2015) that in combination with the high physical work demand causes a high relative workload (Karvonen et al. 1957), which is shown to increase the risk for mortality (Holtermann et al. 2009) and cardiovascular disease (Krause et al. 2007).

Theoretically, improving capacity, e.g. cardiorespiratory fitness, will lower the relative workload and thereby the perceived exertion of the individual (Karvonen et al. 1957). To increase cardiorespiratory fitness, regular aerobic exercise has an established positive effect (Garber et al. 2011). Hence, a period of aerobic exercise has shown to decrease the rating of perceived exertion (RPE), when performing a task at the same absolute workload (Ekblom and Goldbarg 1971). Another important factor in job groups exposed to high physical work demands is the need for recovery (NFR) after a workday, which is highest in job groups experiencing high levels of time pressure and physical work demands (Sluiter 1999). Thus, RPE and NFR are relevant aspects in effect evaluation of aerobic exercise interventions among cleaners and are hypothesised to decrease following an intervention.

Another relevant aspect is work ability that is shown to be diminished when the physical work demand is high (Sell et al. 2009). However, the effects of aerobic exercise interventions on work ability are inconsistent (Pohjonen and Ranta 2001; Nurminen et al. 2002; Jørgensen et al. 2011). One study detected no change at 12 month follow-up after a 9 months of aerobic exercise (Pohjonen and Ranta 2001), whereas another study found a significant improvement in work ability following 8 months of aerobic exercise (Nurminen et al. 2002). Among Danish cleaners, a previous study found no effect of physical exercises or cognitive behavioural training on work ability at 12 month follow-up (Jørgensen et al. 2011). Nonetheless, high work ability is of critical importance for preventing early retirement (Sell et al. 2009) and, therefore, relevant within the cleaning sector continuously cutting back and competing in a global economy, constantly pushing the productivity to its limit. Furthermore, productivity has been proposed to also be affected by aerobic exercise (Pronk et al. 2004). Thus, evaluations of intervention effects on productivity and work ability are of importance (Herod and Aguiar 2006), and we hypothesised that both will increase following an intervention of aerobic exercise.

The main outcome of the current study, cardiorespiratory fitness, has previously been reported. (Korshøj et al. 2015). In short, cardiorespiratory fitness increased alongside a decrease in aerobic workload, as well as resting- and sleeping heart rate after 4 months of aerobic exercise. However, the study did not report on a number of outcomes that have been identified as important factors for future health and employability. Thus, the aim of the present study was to investigate the 4 and 12 months effects on the secondary outcomes of work ability, self-reported productivity, RPE and NFR, from a worksite aerobic exercise intervention among cleaners.

## Methods

### Study design

**Fig. 1:**
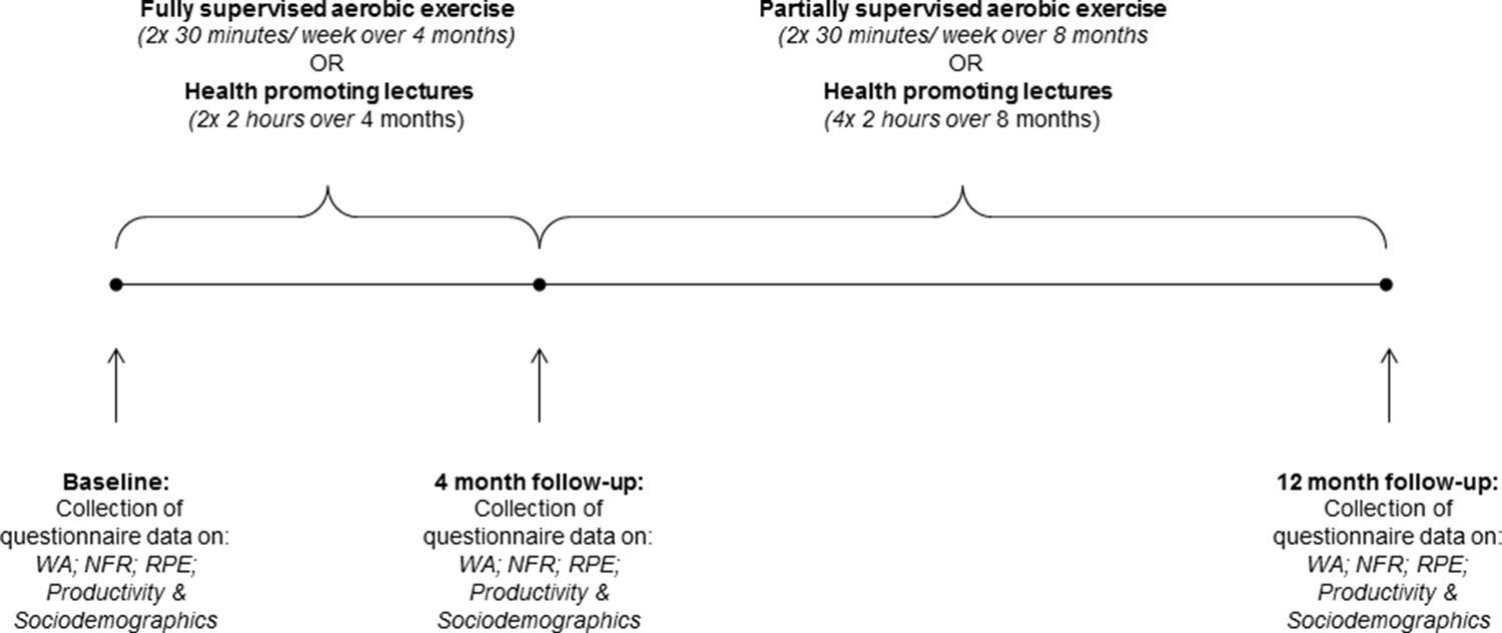
Overview of the study design. *WA* work ability, *NFR* need for recovery, *RPE* rating of perceived exertion

The study was conducted as a cluster-randomised controlled trial, as previously described (Korshøj et al. 2012). In short, the study evaluated the effects of an aerobic exercise worksite intervention over a period of 12 months among cleaners. All outcomes reported were collected at baseline as well as 4 and 12 months after baseline. The study design is illustration in Fig. 1. All participants were informed about the purpose and content of the study and gave their written informed consent prior to participation in accordance with the Declaration of Helsinki. The study was approved by the Danish Data Protection Agency and the Ethics Committee for the regional capital in Denmark (journal number H-2-2011-116), and registered as ISRCTN86682076 in Current Controlled Trials.

### Participants

Cleaning companies located in the Greater Copenhagen area of Denmark were invited to participate in the study. The detailed company recruitment procedure is described elsewhere (Korshøj et al. 2012). Inclusion criteria for enrollment in the current study were employment as a cleaning assistant for more than 20 h per week and aged 18‒65 years. Pregnancy excluded from any participation.

### Data collection

Data were collected via a health check conducted at baseline as well as 4 and 12 months after baseline. The health check consisted of a structured interview with validated questions concerning work ability, NFR, RPE, and self-rated productivity. Further, information about age, sex, length of service, smoking status, as well as level of physical activity in work and leisure time was collected (Korshøj et al. 2012). In addition, objective physical measures of weight (Tanita BC418, Tokyo, Japan) and height (Seca model 213 1721009, Hamburg, Germany) were also collected.

### Randomisation and intervention

The participants were randomised to either an aerobic exercise group or a reference group receiving lectures. In short, the randomisation was performed at cluster level, with clusters set within strata. Stratums were according to nearest manager of the participant. Clusters were balanced on geographical work location, sex, age, and length of service. Clusters were paired with respect to number of participants, sex, age, and length of service. Each cluster pairing was alternately allocated to the two intervention groups depending on the flip of a coin (Korshøj et al. 2012).

Participants randomised to aerobic exercise were offered 2 weekly aerobic exercise sessions of 30 min over the entire 12 month intervention period. All aerobic exercise sessions during the initial 4 months were supervised by a member of the research team, with the supervision gradually declining through the remaining 8 months. The aerobic exercise sessions targeted an average intensity of minimum 60% of maximal oxygen consumption. The content of the aerobic exercise sessions were determined through a modified intervention mapping approach involving researchers as well as management and employee representatives from the participating companies. The main focus points were to make the aerobic exercise sessions feasible for the company, and motivating for the participants. As a result the content was individually tailored for each of the participating companies, but included aerobics, treadmill running and indoor biking. The lectures offered to the reference group addressed a range of topics related to healthy living, but not physical activity. Both the aerobic exercise sessions and the lectures were offered during paid working hours (Korshøj et al. 2012).

### Questionnaire

To assess work ability, we used the single item ‘current work ability compared with the lifetime best’ from the original Work Ability Index (WAI) (Tuomi et al. 1998). The item had numeric values ranging from 0 (worst work ability ever) to 10 (best work ability ever). This item has previously been shown to highly correlate with the entire WAI score (Ilmarinen and Lehtinen 2004).

Self-rated productivity was determined using the item ‘On a scale from 0 to 10 where 0 is the worst job performance anyone could have at your job and 10 is the performance of a top worker how would you rate your overall job performance on the days you worked during the past 4 weeks (28 days)?’ from the WHO Health and Work Performance Questionnaire (Kessler et al. 2003). The question focuses on the overall work performance of the employee. The item had numeric values ranging from 0 (worst work performance ever) to 10 (best work performance ever).

A modified version of the need for recovery after work scale (van Veldhoven and Meijman 1994; van Veldhoven and Broersen 2003) using five of the original 11 statements were used to determine need for recovery: ‘I find it hard to relax at the end of a working day’; ‘At the end of a working day I am really feeling worn-out’; ‘After the evening meal, I generally feel in good shape’; ‘When I get home from work, I need to be left in peace for a while’; and ‘Often, after a day’s work I feel so tired that I cannot get involved in other activities’. Each statement had five response categories ranging from “never” to “always”. These categories were assigned a numeric value ranging from 1 (never) to 5 (always) and a scoring index was determined by the mean score for all five statements (Garde et al. 2012). Finally, the mean score was converted into a scale from 0 to 100, with 100 representing the greatest need for recovery (de Croon et al. 2006). These five statements have previously been shown to highly correlate with the entire NFR score (Garde et al. 2012).

The Borg’s scale for physical exertion were used to determine RPE by asking ‘How physically strenuous do you consider your current job?’, with numeric values ranging from 6 (very, very light) to 20 (very, very strenuous) (Borg 1970).

### Sample size

The A-prior power calculation for the study is based on cardiorespiratory fitness as the primary outcome and reported in the clinical trial. To show an expected increase of 4% in cardiorespiratory fitness would require 52 participants in each of the two intervention groups to show significance at a level of 0.05%. The expected increase in cardiorespiratory fitness is based on an expected drop out of participants of 30%. No A-prior power calculation was conducted for work ability, self-reported productivity, RPE, or NFR.

### Statistical analysis

Statistical analyses were performed using the IBM SPSS statistics software (version 21) (Armonk, NY, USA) and the SAS statistical software for Windows (version 9.3) (Cary, NC, USA).

All analyses were performed according to the intention-to-treat principle (ITT), in which all randomised participants are included in the statistical analyses (Lachin 2000). Missing values were not imputed either for outcome or covariate variables (Altman 2009). Both within- and between-group 4 and 12 month changes of all outcomes were computed with standard errors and 95% confidence intervals. Differences in 4 and 12 month changes of all outcomes were analysed in a linear repeated-measures 2 × 2 mixed-model. Independent categorical variables (fixed factors) were group (aerobic exercise and reference), measurement time (baseline, 4 and 12 month follow-up), and the interaction between group and measurement time. Participants were entered into the model as a random effect nested in clusters to account for the cluster-based randomisation. Covariates were chosen based on baseline differences between groups on theoretically considered confounders and their statistical association with the group and measurement time. Of the selected covariates, only age shows a significant influence on RPE at 4 months. A further model adjusted for the interaction between intervention × age was non-significant for all outcomes.

The following covariates were incrementally taken into account in the analysis (reference value in parenthesis): model 1—baseline value of the respective outcome; model 2—additionally adjusted for age, sex (male), smoking status (never smoked and/or currently non-smoking), length of service and level of leisure time physical activity (< 2 h per week light activity).

Pearson correlation coefficient was determined to identify any potential correlation between adherence to the aerobic exercise and work ability, productivity, NFR and RPE, respectively.

Body mass index (BMI) was calculated as body weight (kg)/body height (m^2^).

Two secondary between-group analysis stratified on age (median spilt by 45 years of age) and adherence to the aerobic exercise (</> 50%) were conducted.

## Results

### Flow of participants

**Fig. 2:**
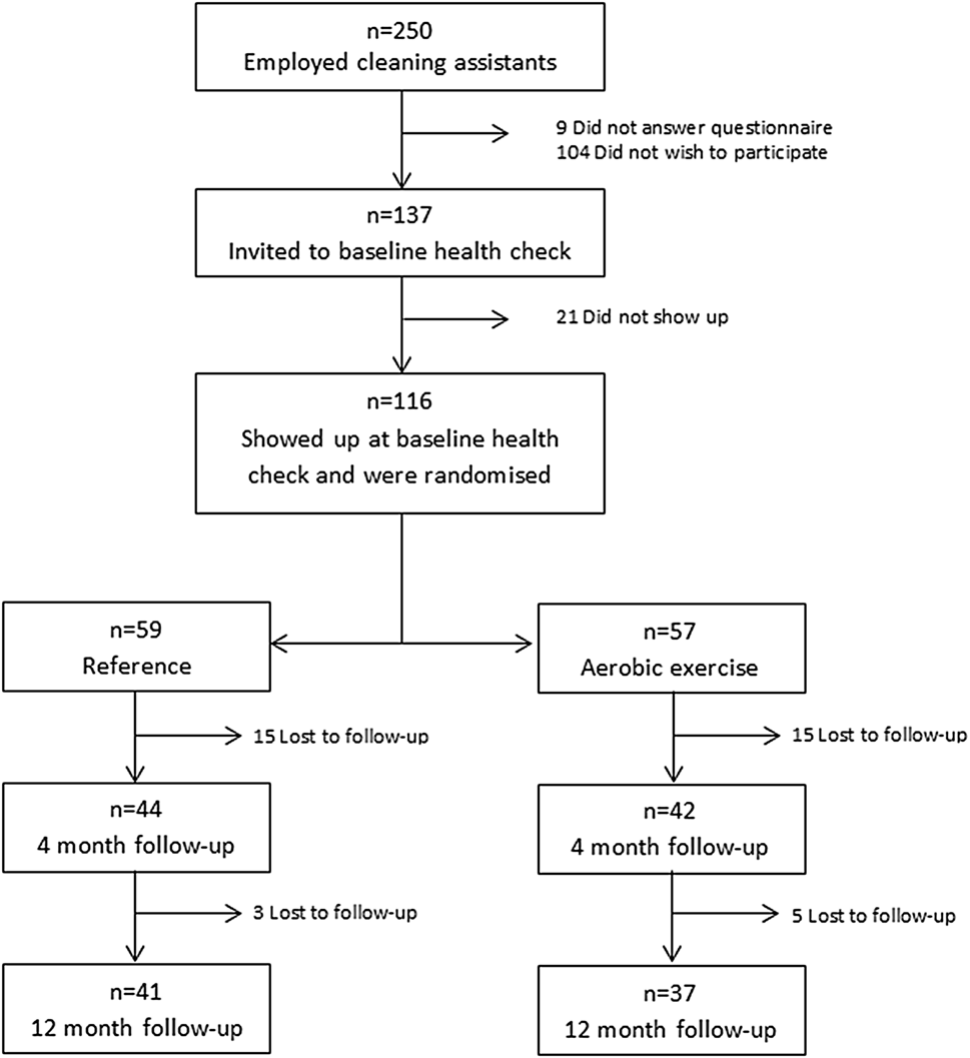
Flow of the participants

All three contacted companies agreed to participate. The details of the participant flow are presented in Fig. 2. In brief, 250 cleaning assistants were invited to participate, out of which 116 were randomised. A total of 30 (26%) and 38 (33%) participants were lost to follow-up at 4 and 12 month follow-up, respectively.

The following significant (*p* < 0.05) differences were seen between the consenters and non-consenters: the consenters had less length of service and were more frequently born outside Denmark. No differences were seen in diagnosed illnesses, levels of occupational and leisure time physical activity, sex, age, height and weight.

The main self-reported reasons for non-participation among non-consenters were lack of time (40%) and not finding the study relevant (11%) (Korshøj et al. 2015). Of the consenters, 21 were not randomised because they withdrew from the study before the baseline measurement took place or did not show up at the baseline measurement. Those consenters being randomised were more frequently born outside Denmark (*p* < 0.05) than the consenters not being randomised.

### Compliance

During the 4 month intervention, the participants randomised to aerobic exercise participated on average in 51% of the planned sessions, including five participants with zero adherences. Participants not lost to follow-up participated in 64% of the planned sessions, none with zero adherences. The reasons for missed sessions in the aerobic exercise group were as follows: vacation and days off work (52%); medical appointments; hospitalisation and/or sick leave (30%); and meetings at work, courses and/or urgent job tasks (18%). After every fourth week of the 4 month intervention, heart rate was monitored during the aerobic exercise session, yielding an average heart rate reserve of 67 ± 13%. Overall, 94% of the planned sessions were offered as planned (Korshøj et al. 2015).

Adherence during the final 8 months of the intervention was not recorded because not all sessions were supervised.

Secondary analyses showed no correlation between neither of the outcomes and percentage of adherence (</> 50%) to the aerobic exercise sessions from baseline to 4 month follow-up.

### Baseline characteristics of the study population

The baseline characteristics of the study population are presented in Table 1. In short, the entire study population of 116 cleaners consisted of 75.9% females and was on average 45.3 years old, with a BMI of 26.7 kg/m^2^, with a length of service of 11.9 years, and 24.1% were current smokers. No significant differences were observed between the intervention groups.

**Table 1:** Description of the randomised study population of cleaners at baseline (*N* = 116), stratified by intervention group

	Aerobic exercise (*n* = 57)			Reference (*n* = 59)			*p*
	Mean	SD	*n*	Mean	SD	*n*	
Age (years)	44.9	9.2		45.7	8.1		> 0.05
Sex (% females)	75.4		43	76.3		45	> 0.05
Height (m)	1.63	0.09		1.62	0.08		> 0.05
Weight (kg)	69.7	12.7		71.7	15.4		> 0.05
BMI (kg/m^2^)	26.2	4.0		27.1	4.9		> 0.05
Length of service (years)	12.3	8.7		11.5	6.8		> 0.05
Current smoker (%)	22.8		13	25.4		15	> 0.05
Leisure time physical activity (% < 2 h/week light activity or light activity 2‒4 h/week)	78.9		45	66.1		39	> 0.05
Physical activity at work (% of standing/walking work, including lifting and strenuous physical work)	63.2		36	57.6		34	> 0.05
Cardiorespiratory fitness (mlO_2_ min^−1^ kg^−1^)	24.8	5.8		25.0	7.2		> 0.05
Work ability (scale 0–10)	9.35	1.72		9.19	1.65		> 0.05
Productivity (scale 0–10)	9.12	2.13		9.37	1.82		> 0.05
Need for recovery (scale 0–100)	53.9	28.0		61.4	25.4		> 0.05
Rating of perceived exertion (scale 6–20)	12.9	3.2		13.1	3.3		> 0.05

Within the aerobic exercise group, some significant differences (*p* ≤ 0.05) between those participants being randomised (*n* = 57) and those lost to follow-up after 12 months (*n* = 20) were found. Those lost to follow-up at 12 months had significantly less length of service (5 years), a significantly higher proportion stated to be current smokers (25%) and there was a tendency (*p* = 0.053) towards a higher proportion stating their occupational physical activity as strenuous (29%). No differences were seen within the reference group.

### Intervention effects at 4 month follow-up

Table 2 shows the within- and between-group differences in change score at 4 month follow-up for the fully adjusted model.

**Table 2:** Within-group means baseline to 4 month follow-up for each group, between-group difference (mean ± standard error) from and percentage change relative to the baseline mean of the randomised population of cleaners (*N* = 116)

	Aerobic exercise		Reference		Δ	%	SE	95% CI	*p*	*n*
	Baseline	4 months	Baseline	4 months						
Work ability	9.32	9.33	9.27	9.27	0.06	0.65	0.26	− 0.46 to 0.58	0.83	111
Productivity	9.04	9.37	9.09	9.20	0.18	1.95	0.28	− 0.38 to 0.73	0.53	111
Need for recovery	56.1	53.8	59.1	58.4	− 4.6	− 7.9	3.8	− 12.0 to 2.9	0.23	110
Rating of perceived exertion	7.83	7.98	8.00	8.69	− 0.70	− 5.38	0.36	− 1.41 to 0.00	0.05	111

Between-group 95% confidence interval and level of significance are reported. *n* differs between the different outcomes due to missing observations in covariate and/or outcome variables. All outcomes are adjusted for baseline value of the respective outcome, age, sex (male), smoking status (never smoked and/or currently non-smoking), length of service and level of leisure time physical activity (< 2 h per week light activity)

No significant between-group difference in change score was found at 4 month follow-up. RPE showed a tendency towards a between-group decrease of − 0.70 ± 0.36 (95% CI − 1.41 to 0.00, *p* 0.05).

### Intervention effects at 12 month follow-up

Table 3 shows the within- and between-group differences at 12 month follow-up for the fully adjusted model. The results reported are changes in the aerobic exercise group compared to the reference group.

**Table 3:** Within-group means pre and post intervention, between-group difference (mean ± standard error) from baseline to 12 month follow-up and percentage change relative to the baseline mean of the randomised population of cleaners (*N* = 116)

	Aerobic exercise		Reference		Δ	%	SE	95% CI	*P*	*n*
	Baseline	12 months	Baseline	12 months						
Work ability	9.23	9.60	9.21	9.01	0.59	6.36	0.27	0.05 to 1.13	0.03	102
Productivity	9.06	8.66	9.12	8.57	0.09	0.97	0.33	− 0.57 to 0.74	0.79	102
Need for recovery	56.7	49.6	60.0	60.6	− 11.0	− 19.0	4.5	− 19.8 to − 2.2	0.02	102
Rating of perceived exertion	8.13	8.32	8.21	8.70	− 0.39	− 3.00	0.36	− 1.10 to 0.32	0.28	102

Between-group 95% confidence interval and level of significance are reported. All outcomes are adjusted for baseline value of the respective outcome, age, sex (male), smoking status (never smoked and/or currently non-smoking), length of service and level of leisure time physical activity (< 2 h per week light activity)

At 12 month follow-up, a significant between-group difference was found for work ability and NFR. An increase in between-group difference was seen in work ability 0.59 ± 0.27 (95% CI 0.05–1.13, *p* 0.03), and a decrease in between-group difference was seen in NFR − 11.0 ± 4.5 (95% CI − 19.8 to − 2.2, *p* 0.02).

**Fig. 3:**
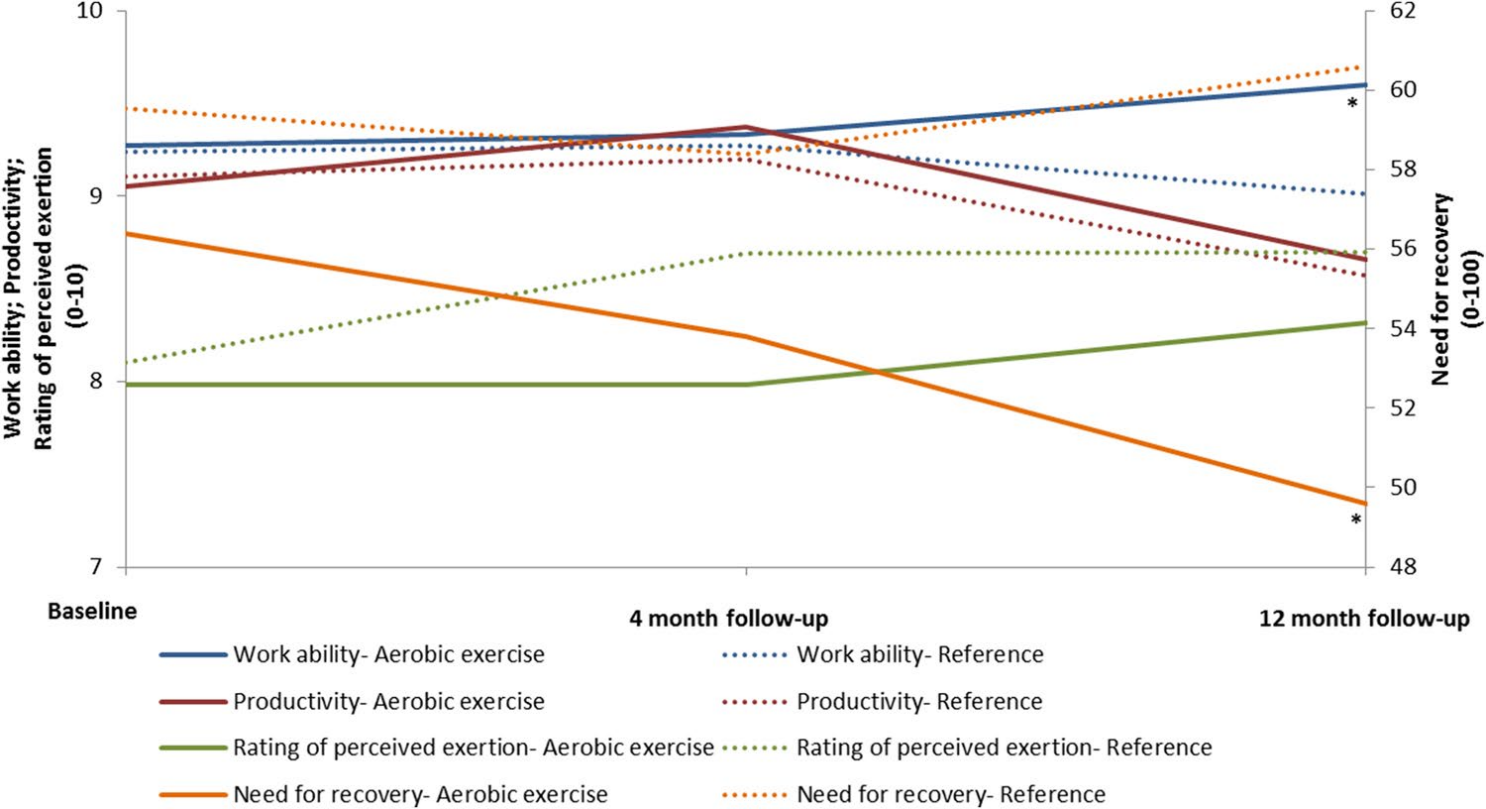
Development in the work ability, productivity, rating of exertion, and need for recovery during the intervention period. Work ability, productivity, and rating of exertion are presented on a scale from 0 to 10 (primary vertical axis). Need for recovery is presented on a scale from 0-100 (secondary vertical axis). *p < 0.05 significant change between baseline and 12 month follow-up in the aerobic exercise group compared to the reference group

Figure 3 illustrates the development in the outcomes over the intervention period.

### Sensitivity analysis

Table 4a and b shows the effect of the exercise intervention for all four outcomes stratified on age from baseline to 4 and 12 months, respectively. At 4 months, productivity and RPE were significantly influenced by age (≤ 45 years). At 12 months, all outcomes were significantly influenced by age (≤ 45 years).

**Table 4:** Between-group difference (mean ± standard error) at (a) 4 months and (b) 12 months stratified by median age of the study population (≤ 45 years)

(a) 4 months	Older (> 45 years)					Younger (≤ 45 years)				
	Δ	SE	95% CI	*p*	*n*	Δ	SE	95% CI	*p*	*n*
Work ability	− 0.34	0.41	− 1.16 to 0.48	0.41	53	0.61	0.37	− 0.12 to 1.33	0.10	58
Productivity	− 0.44	0.46	− 1.36 to 0.47	0.34	53	0.99	0.37	0.26 to 1.72	0.009	58
Need for recovery	− 0.4	5.3	− 11.0 to 10.3	0.95	52	− 9.8	6.0	− 21.7 to 2.1	0.11	58
Rating of perceived exertion	− 0.33	0.54	− 1.40 to 0.73	0.53	53	− 1.13	0.54	− 2.20 to – 0.07	0.04	58

(b) 12 months	Older (> 45 years)					Younger (≤ 45 years)				
	Δ	SE	95% CI	*p*	*n*	Δ	SE	95% CI	*p*	*n*
Work ability	0.18	0.43	− 0.67 to 1.02	0.68	49	1.12	0.37	0.39 to 1.85	0.003	53
Productivity	− 0.92	0.57	− 2.04 to 0.21	0.11	49	1.27	0.38	0.52 to 2.03	0.001	53
Need for recovery	− 2.8	7.0	− 16.8 to 11.1	0.69	48	− 23.5	6.4	− 36.2 to − 10.8	0.0004	53
Rating of perceived exertion	− 0.22	0.56	− 1.33 to 0.88	0.69	49	− 0.26	0.53	− 1.31 to 0.79	0.63	53

Between-group 95% confidence interval and level of significance are reported. All outcomes are adjusted for baseline value of the respective outcome, age, sex (male), smoking status (never smoked and/or currently non-smoking), length of service and level of leisure time physical activity (< 2 h per week light activity)

## Discussion

This study showed both increased work ability and reduced NFR as result of 2 weekly aerobic exercise sessions over a period of 12 months among cleaners. In contrast, the intervention did not positively or negatively affect RPE and self-rated productivity at 12 month follow-up. At 4 month follow-up, no significant effects were shown. Further, an analysis stratified on median age (45 years) showed that those in the aerobic exercise group below 45 years of age achieved significant beneficial effects from the intervention, compared to those in the reference group below 45 years of age. Similar effects were not seen among participants above 45 years of age.

### Work ability

In the current study, work ability was improved after 12 months of aerobic exercise among cleaners. However, a period of 4 months seemed insufficient to affect work ability. The results support the findings from a recent study showing an improved work ability in a population partly consisting of blue-collar workers after 6 months of aerobic exercise, which was still present after 30 months (Rutanen et al. 2014). Despite the presence of a high initial work ability level, the present study improved the work ability of the cleaners. This is surprising, as having high initial work ability level increases the possibility of being affected by the existence of a potential ceiling effect. Further, another study, offering aerobic exercise to a working population having a high initial level of work ability showed no effect on work ability (Blangsted et al. 2008). In this study, the participants within the aerobic exercise group improved their work ability by 4.0%, thereby showing an improvement similar to the 5.4% enhancement in work ability found by Rutanen and colleagues. This is an important finding because work ability previously has been identified as a predictor for long-term sickness absence and early retirement (Sell et al. 2009), and this increase of 6.4% between groups might, therefore, have a positive long-term influence on both sickness absence and early retirement.

### Need for recovery

The aerobic exercise intervention decreased NFR after 12 months of aerobic exercise. Previously, a positive association between the amount of self-reported leisure time physical activity and NFR has been shown (Sonnentag 2001). As far as the authors know, only one previous study exists that reported decreased NFR as a result of a worksite exercise intervention among hospital workers (Strijk et al. 2012). However, this intervention comprised a mix of yoga and aerobic exercise. As a result, the current study may be the first to report decreased NFR as a result of an intervention solely comprising aerobic exercise among blue collar workers. As a result of the intervention, the participants within the aerobic exercise group experienced a considerable decrease in NFR of 12.5%, which is similar to the findings by Strijk and colleagues (Strijk et al. 2012) showing an 11.2% decrease in NFR. Decreasing the NFR by more than 10% is thought to lower the diurnal blood pressure (Stewart et al. 2006), sickness absence (de Croon et al. 2003), and cardiovascular disease risk (van Amelsvoort 2003).

However, no significant change in NFR after the first 4 months of aerobic exercise was seen, only after 12 months. In the study by Strijk and colleagues (Strijk et al. 2012), the combined yoga and aerobic exercise intervention showed decreased NFA after 6 months. This may suggest that a period of more than 4 months of aerobic exercise is needed to decrease the NFR. Furthermore, change in NFR is affected by adherence to the intervention and often a high adherence is required to achieve a lowered NFR (Strijk et al. 2012). The adherence to the aerobic exercise in the current study was 64% for the initial 4 months, which suggests that any decrease in NFR within 4 months would require an adherence of minimum 64%. As a result of the study design, adherence was not monitored for the remaining 8 months. However, as a decreased NFR was found at 12 month follow-up (Table 3), the participants in the aerobic exercise group must have obtained a sufficient adherence during the final 8 months to cause the decreased NFR.

### Rating of perceived exertion

No change in RPE was found after neither 4 nor 12 months of intervention, even though a tendency towards a decreased RPE was observed at 4 months of follow-up (Table 2). The effect of introducing aerobic exercise in job groups with high physical work demands is to some extent unknown, but it has previously been speculated that weekly exercise may act as a physical stressor causing physiological overload on the cleaners, who already are exposed to a high physical work demand (Korshøj et al. 2015). A possible explanation for the unchanged RPE could be that performing an aerobic exercise during working hours introduces both a physical and mental stress, as the participants need to carry out the same amount of work in a shorter period of time, which counteracts the potential beneficial effects of aerobic exercise on RPE. On the other hand, the unaltered RPE also indicate that the participants were not more exhausted by replacing cleaning work tasks in 2 × 30 min/week by aerobic exercise.

### Productivity

The present study did not affect self-rated productivity after neither 4 nor 12 months of intervention. Classically, an improved self-rated productivity is observed after a period of aerobic exercise (Rosenfeld et al. 1989). However, a later review has concluded that no evidence for a positive effect of aerobic exercise on productivity exists (Proper et al. 2002). This conclusion has been supported by more recent studies showing that productivity was unaffected by physical exercise for a period of 12 months (Blangsted et al. 2008). Nevertheless, a recent study shows improved productivity as a result of physical exercise as part of a workplace health program (von Thiele and Hasson 2011). However, compared to the current study the time spent on physical exercise on a weekly basis was more than double (von Thiele and Hasson 2011), implying that the duration of exercise per week may play a significant role with respect to changes in productivity. In contrast, a study on health care workers showed no relationship between an increased fitness level and productivity, while increased muscle strength significantly increased productivity (Christensen et al. 2015). Then again, productivity did not decrease as a result of the intervention, which hereby shows that the positive effects on work ability and NFR can be achieved without any loss of productivity, even though the company needs to invest time when implementing an aerobic exercise program.

### The influence of age

The results from the age stratified analysis suggest that there is a tendency towards that the younger individuals experience better effects from participating in the training intervention on productivity and RPE after 4 months, as well as work ability, productivity, and NFR after 12 months. This finding indicates that the beneficial results on work ability and NFR to some extent are driven by a larger responsiveness to the intervention among the younger workers. However, because no significant interaction for age was found, and age was included as a confounder in the adjusted analysis, no conclusion on a strictly age-dependent effect of the intervention can be drawn. This issue needs to be investigated in future studies.

### Strengths and limitations

Overall, the study builds on sound methodology using a cluster-randomised controlled trial design. Furthermore, all data analysis is performed in accordance with the intention-to-treat principle and using a mixed-model, which both increases the amount of data used and allows for sophisticated handling of missing observations (Lachin 2000). Linear repeated-measures mixed-model is a robust and often used model with respect to handling missing values in intervention studies. However, even though the model handles missing values, it might also lead to potential bias of the results which ought to be taken into consideration in the interpretation of the results.

In addition, there are a number of limitations associated with the study. Due to the design of the aerobic exercise intervention, the researchers were prevented from controlling the intensity of, as well as the adherence to, the aerobic exercise for the past 8 months of the intervention.

The dropout rate at 4 month follow-up was 29%, which was within the predefined level of acceptance according to the power calculations (Korshøj et al. 2012). However, at 12 month follow-up, the dropout rate had increased to 38%. During a worksite intervention lasting for a period of 12 months, a certain dropout must be expected due to factors such as job turnover and long-term sickness absence. Further, cleaners are an occupational group with a high labour turnover (Wills 2008). As a result, the observed dropout is within a level that would be expected based on normal conditions within cleaning industry (Jørgensen et al. 2011). In combination with the differences in demographics shown between completers and dropouts, the increased dropout leads to a selection bias towards a more healthy population being analysed, causing the generalisability of the study to the entire population of cleaners to decrease.

The question used for self-rated productivity may not be suitable in this population of cleaners. All participants were subject to centrally administered work plans and were therefore forced to clean specific areas within a predefined timeframe regardless of intervention group. The chosen productivity question may, therefore, not have been sufficiently specific since the participants did not have an opportunity to perform more work. As a result, a potential improvement in productivity could not be detected.

The inability to blind the participants, which is a general problem in studies with a designated training group, introduces multiple risks of non-specific effects, including possible placebo effects in respect of changes in the selected outcomes, as well as the possibility of a Hawthorne effect (Landsberger 1958). In combination with the use of self-reported questionnaire data, there is a risk of overestimating potential effects of the intervention, as the participants expect a positive outcome. In order to minimise the risk of overestimating any potential effects of the intervention, it was emphasised to include the reference group as much as possible as an active part of the worksite intervention. In addition, the tendencies in the self-reported data are in accordance with the objectively measured improvements like cardiorespiratory fitness and reduced heart rate reserve at work previously reported from the study (Korshøj et al. 2015), which supports that the findings from the current study are genuine. Furthermore, there is a risk of recall bias when using self-reported data. However, the risk of recall bias would be expected to be identical for both intervention groups, thereby decreasing the possibility of detecting significant differences between the intervention groups.

An increased risk of Type 1 error is identified by not performing any kind of correction of the α first error levels. However, performing a correction introduces a substantial risk of Type 2 error. Consequently, the effects of the intervention are being evaluated through a critical interpretation of the p-values.

## Conclusion

A 12 month worksite aerobic exercise intervention among cleaners improved work ability and NFR, but had no effect on RPE and productivity. A period of 4 months of aerobic exercise seemed insufficient to affect the work ability and NRF. A tendency towards a greater effect of the aerobic exercise was seen among the younger individuals. The improved work ability and NFR may have a positive long-term effect on occupational health as well as employability, and giving that the effects are the result of a relatively small amount of aerobic exercise, the findings from the current study suggest that aerobic exercise ought to be considered to be offered as an integrated part of the work day for occupational groups with high physical work demands.

## What this paper adds

- Cleaners are an occupational group with a high physical work demand and high productivity level, increasing their risk of early retirement and sickness absence.
- The effect of worksite interventions, including aerobic exercise among cleaners on risk factors for early retirement and sickness absence, like work ability, need for recovery, productivity and rating of exertion, is largely unknown.
- In this study, work ability and need for recovery were improved following an intervention with 2 × 30 min of aerobic exercise per week during paid working hours, for a period of 12 months.
- A period of 4 months of aerobic exercise was insufficient to change work ability, need for recovery, productivity and rating of exertion, despite a relative high intensity and adherence to the aerobic exercise sessions.
- Companies employing occupational groups with high physical work demands should seek to incorporate aerobic exercise, especially targeting older employees, as a part of the work day to improve occupational health and employability.
